# Gut Microbiota Composition Is Associated With the Global DNA Methylation Pattern in Obesity

**DOI:** 10.3389/fgene.2019.00613

**Published:** 2019-07-03

**Authors:** Bruno Ramos-Molina, Lidia Sánchez-Alcoholado, Amanda Cabrera-Mulero, Raul Lopez-Dominguez, Pedro Carmona-Saez, Eduardo Garcia-Fuentes, Isabel Moreno-Indias, Francisco J. Tinahones

**Affiliations:** ^1^Deparment of Endocrinology and Nutrition, Virgen de la Victoria University Hospital, Institute of Biomedical Research in Malaga (IBIMA) and University of Malaga, Malaga, Spain; ^2^CIBER Physiopathology of Obesity and Nutrition (CIBERobn), Institute of Health Carlos III, Madrid, Spain; ^3^Bioinformatics Unit, Centre for Genomics and Oncological Research: Pfizer/University of Granada/Andalusian Regional Government, PTS, Granada, Spain; ^4^Department of Gastroenterology, Virgen de la Victoria University Hospital, Institute of Biomedical Research in Malaga (IBIMA) and University of Malaga, Malaga, Spain

**Keywords:** obesity, gut microbiota, methylation, epigenetics, adipose tissue

## Abstract

**Objective:** Obesity and obesity-related metabolic diseases are characterized by gut microbiota and epigenetic alterations. Recent insight has suggested the existence of a crosstalk between the gut microbiome and the epigenome. However, the possible link between alterations in gut microbiome composition and epigenetic marks in obesity has been not explored yet. The aim of this work is to establish a link between the gut microbiota and the global DNA methylation profile in a group of obese subjects and to report potential candidate genes that could be epigenetically regulated by gut microbiota in adipose tissue.

**Methods:** Gut microbiota composition was analyzed in DNA stool samples from 45 obese subjects by 16S ribosomal RNA (rRNA) gene sequencing. Twenty patients were selected based on their Bacteroidetes-to-Firmicutes ratio (BFR): HighBFR group (BFR > 2.5, *n* = 10) and LowBFR group (BFR < 1.2, *n* = 10). Genome-wide analysis of DNA methylation pattern in both whole blood and visceral adipose tissue of these selected patients was performed with an Infinium EPIC BeadChip array-based platform. Gene expression analysis of candidate genes was done in adipose tissue by real-time quantitative PCR.

**Results:** Genome-wide analysis of DNA methylation revealed a completely different DNA methylome pattern in both blood and adipose tissue in the low BFR group vs. the high BFR group. Two hundred fifty-eight genes were differentially methylated in both blood and adipose tissue, of which several potential candidates were selected for gene expression analysis. We found that in adipose tissue, both *HDAC7* and *IGF2BP2* were hypomethylated and overexpressed in the low BFR group compared with the high BFR group. β values of both genes significantly correlated with the BFR ratio and the relative abundance of Bacteroidetes and/or Firmicutes.

**Conclusions:** In this study, we demonstrate that the DNA methylation status is associated with gut microbiota composition in obese subjects and that the expression levels of candidate genes implicated in glucose and energy homeostasis (e.g., *HDAC7* and *IGF2BP2*) could be epigenetically regulated by gut bacterial populations in adipose tissue.

## Introduction

Obesity has reached a pandemic scale worldwide, mainly caused by changes in lifestyles that include regular consumption of high-calorie food and a critical reduction of physical activity. Emerging evidence suggests that an altered composition and diversity of gut microbiota could play an important role in the development of obesity and related metabolic disorders such as type 2 diabetes (T2D) or non-alcoholic fatty liver disease (Cani, 2013; Han and Lin, 2014; Moreno-Indias et al., 2014; Leung et al., 2016; Cani, 2019). The relative amount of the two dominant phyla in gut microbiota, Firmicutes and Bacteroidetes, is altered in obesity conditions both in humans and in animal models (Ley et al., 2005; Ley et al., 2006; Turnbaugh et al., 2006; Verdam et al., 2013). Besides, the Bacteroidetes-to-Firmicutes ratio (BFR) has been widely associated with the inflammatory and metabolic state in obesity (Cani et al., 2009; de La Serre et al., 2010; Verdam et al., 2013). Several mechanisms have been proposed as a link between obesity and gut microbiota, for instance, the production of microbial metabolites that regulate energy metabolism, metabolic endotoxemia, or the modulation of the secretion of hormones by intestinal cells (Cani, 2019).

Epigenome captures environmental and lifestyle events. Recent insight has suggested a role of epigenetics in the development of obesity and related metabolic disorders (van Dijk et al., 2015; Davegardh et al., 2018). More recently, the existence of a crosstalk between the gut microbiome and the epigenome has been suggested (Qin and Wade, 2018). It has been proposed that certain metabolites generated by the gut microbiota such as short-chain fatty acids (SCFAs), folate, and polyamines can act as epigenetic modulators by affecting DNA methylation and inducing histone modifications (Crider et al., 2012; Paul et al., 2015; Bhat and Kapila, 2017; Soda, 2018; Cuevas-Sierra et al., 2019; Ramos-Molina et al., 2019). However, the possible link between alterations in gut microbiome composition and epigenetic marks in the context of obesity has been not explored yet.

In this work, we have established a link between the gut microbiota and the global DNA methylation profile in a group of obese subjects by integrating 16S rRNA gene sequence analysis and epigenome-wide association studies, and we have reported potential candidate genes that could be epigenetically regulated by gut microbiota in adipose tissue.

## Material and Methods

### Study Participants

This is a cross-sectional analysis of 45 morbidly obese subjects [body mass index (BMI) > 40 kg/m^2^] who were consecutively recruited at the Virgen de la Victoria University Hospital for bariatric surgery (Malaga, Spain) from 2015 to 2017. All participants provided written informed consent, and the study protocol and procedures were approved according to the ethical standards of the Declaration of Helsinki by the Research Ethics Committees from all the participating institutions.

### Laboratory Measurements

Blood samples were obtained from the antecubital vein and placed in vacutainer tubes after an overnight fast. The serum was separated by centrifugation for 15 min at 4,000 rpm at 4°C and frozen at −80°C until analysis. Enzymatic methods (Randox Laboratories Ltd). were employed to analyze the levels of serum cholesterol, triglycerides, HDL-cholesterol, glucose, and glycosylated hemoglobin (HbA1c) using a Dimension Vista autoanalyzer (Siemens Healthcare Diagnostics). Serum insulin levels were measured by immunoassay using an ADVIA Centaur autoanalyzer (Siemens Healthcare Diagnostics). Insulin resistance (IR) was calculated from the homeostasis model assessment of IR (HOMA-IR) with the following formula: HOMA-IR = [fasting serum insulin (μU/ml) × fasting blood glucose (mmol/L)]/22.5.

### Gut Microbiota Analysis

Stool samples were collected and immediately frozen at −80°C until DNA extraction. DNA was extracted from fecal samples using the QIAamp DNA Stool Mini Kit (Qiagen, Hilden, Germany) following the manufacturer’s protocol. Ribosomal 16S rRNA gene sequences were amplified from cDNA using the 16S Metagenomics Kit (Thermo Fisher Scientific, Italy). The kit included two primer sets that selectively amplify the corresponding hypervariable regions of the 16S region in bacteria: primer set V2–4–8 and primer set V3–6, 7–9. Libraries were created using the Ion Plus Fragment Library Kit (Thermo Fisher Scientific). Barcodes were added to each sample using the Ion Xpress Barcode Adapters kit (Thermo Fisher Scientific). Emulsion PCR and sequencing of the amplicon libraries were performed on an Ion 520 chip (Ion 520^™^ Chip Kit) using the Ion Torrent S5^™^ system and the Ion 520^™^/530^™^ Kit-Chef (Thermo Fisher Scientific) according to the manufacturer’s instructions.

Base calling and run demultiplexing were performed by using Torrent Suite^™^ Server software (Thermo Fisher), version 5.4.0, with default parameters for the 16S Target Sequencing (bead loading ≤ 30, key signal ≤ 30, and usable sequences ≤ 30). Quality sequences were analyzed using QIIME 1.9.1 software. Briefly, the workflow was the following: operational taxonomic units (OTUs) were calculated by clustering sequences at a similarity of 97% with a closed-reference OTU picking approach. The representative sequences were submitted to the UCLUST to obtain the taxonomy assignment and the relative abundance of each OTU using the Greengenes 16S rRNA gene database. OTUs were collapsed to phylum level in order to calculate the BFR. Raw data can be found in the SRA database public repository from NCBI within the BioProject accession number PRJNA539905.

### DNA Methylation Profiling Using Universal Bead Array

Visceral adipose tissue (VAT) was obtained during bariatric surgery. Biopsy samples were washed in physiological saline and immediately frozen at −80°C until analysis. DNA was extracted from blood and VAT using Zymo ZR 96 Quick gDNA kit (Zymo Research Corp., Irvine, CA, USA) following manufacturer’s instructions. After quantification and purity assessment, a total of 500 ng of genomic DNA was bisulfite treated using the ZymoResearch Infinitum HD FFPE Methylation kit (Zymo Research Corp, Irvine, CA, USA) and was purified by DNA-Clean-Up kit (Zymo Research Corp, Irvine, CA, USA). Over 850,000 methylation sites were interrogated with the Infinium Methylation EPIC Bead Chip Kit (Illumina, San Diego, CA, USA) following the Infinium HD Assay Methylation protocol, and raw data (idat files) were obtained from iScan (Illumina) software.

### Methylation Data Analysis

Raw data files (idat files) were processed to derive beta values after background correction and normalization by BMIQ (Teschendorff et al., 2013). The beta value is the ratio of the methylated probe intensity and the overall intensity, which resulted from the sum of methylated and unmethylated probe intensities. The beta value results in a number between 0 and 1, in which a value of zero indicates that all copies of the CpG site in the sample were completely unmethylated and a value of one indicates that every copy of the site was methylated (Du et al., 2010). Differential methylation, gene set enrichment, and pathway analyses were performed using Partek Genomics Suit with Pathway (version 7.0). To complete the analysis, we used EnrichR (https://amp.pharm.mssm.edu/Enrichr/) (Chen et al., 2013) and the analysis of some ontologies such as Online Mendelian Inheritance in Man (OMIM) diseases. Raw data can be found in the GEO database public repository from NCBI within the accession number GSE131461.

### Gene Expression Analysis

Frozen VAT was homogenized with an Ultra-Turrax 8 (Ika, Staufen, Germany). Total RNA was extracted by RNeasy lipid tissue midi kit (QIAGEN Science, Hilden, Germany) and treated with 55 U of RNase-free deoxyribonuclease (QIAGEN Science, Hilden, Germany), following the manufacturer’s instructions. RNA purity and concentration were determined by 260/280 absorbance ratios on a Nanodrop ND-1000 spectrophotometer (Thermo Fisher Scientific Inc., Waltham, MA). Total purified RNA integrity was checked by denaturing agarose gel electrophoresis and SYBR Safe DNA gel staining (Invitrogen). Total RNA was reverse transcribed to cDNA by a high-capacity cDNA reverse transcription kit with RNase inhibitor (Applied Biosystems, Foster City, CA). Quantitative real-time PCR with duplicates was done with the cDNA. The amplifications were performed using a MicroAmpH Optical 96-well reaction plate (Applied Biosystems, Foster City, CA) on an ABI 7500 Fast Real-Time PCR System (Applied Biosystems, Foster City, CA). Commercially available and pre-validated TaqMan^®^ primer/probe sets were used as follows: cyclophilin A (*PPIA*, 4333763), used as endogenous control for the target gene in each reaction; fibroblast growth factor 1 (*FGF1*, Hs01092738_m1); fibroblast growth factor 10 (*FGF10*, Hs00610298_m1); lysine demethylase 4B (*KDM4B*, Hs00392119_m1); interleukin-7 (*IL7*, Hs00174202_m1); insulin-like growth factor 2 mRNA-binding protein 2 (*IGF2BP2*, Hs01118009_m1); histone deacetylase 7 (*HDAC7*, Hs01045864_m1); ER degradation enhancing alpha-mannosidase-like protein 1 (*EDEM1*, Hs00976004_m1); activating transcription factor 6 (*ATF6*, Hs00232586_m1); and cyclin-dependent kinase 6 (*CDK6*, Hs01026371_m1). A threshold cycle (*Ct* value) was obtained for each amplification curve and normalized by subtracting the *Ct* value of the endogenous gene and expressed as Δ*Ct* value and expressed in linear scale as 2^−Δ^ *^Ct^*.

### Statistical Analysis

Continuous variables are summarized as means ± SD or SE. Discrete variables are presented as frequencies and percentages. Differences in clinical characteristics between two groups were analyzed using the Mann–Whitney *U* test. The Spearman correlation coefficients were calculated to estimate the correlations between variables. Statistical analyses were carried out with the statistical software package SPSS version 15.0 (SPSS Inc., Chicago, IL, United States). Values were considered to be statistically significant when the *p* < 0.05. Association analysis between phenotypes and probes was assessed with the R package CpGassoc in R 3.3.3 (Barfield et al., 2012). FDR-corrected *p* < 0.01 was considered statistically significant.

## Results

**Figure 1:**
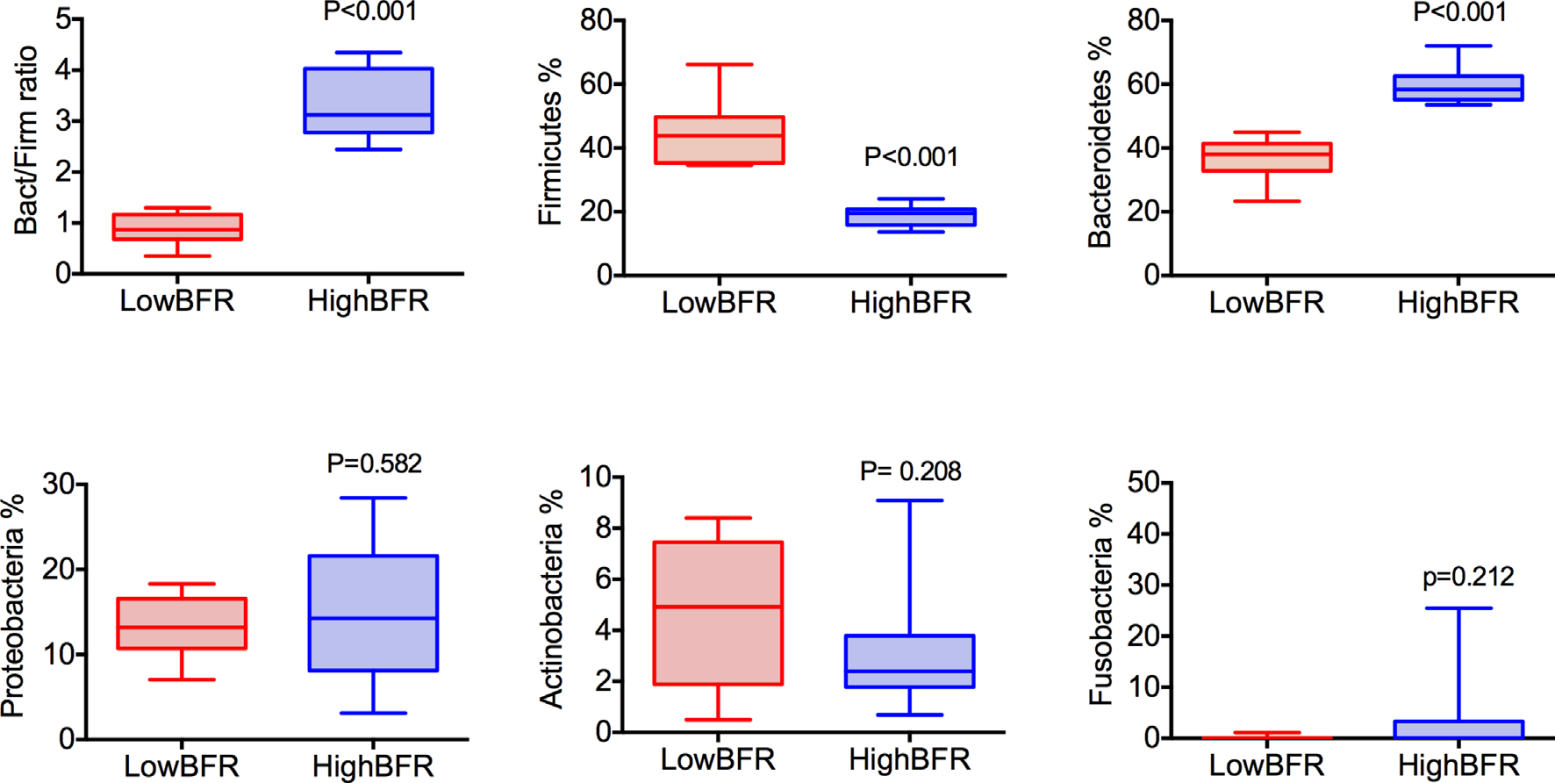
Categorization of study patients into the HighBFR and LowBFR groups according to dominant bacterial phyla. Box plots (range min–max) show the Bacteroidetes-to-Firmicutes ratio and the relative abundance (%) of five more predominant bacterial phyla: Bacteroidetes, Firmicutes, Proteobacteria, Acidobacteria, and Fusobacteria. Comparisons between groups were performed using *t* test analysis (***p < 0.0001).

Analysis of the gut microbiota composition was performed in a group of 45 patients with morbid obesity (**Table S1**). Moreover, to determine the potential contribution of gut microbiota composition to the global DNA methylome, we extracted genomic DNA from whole blood and VAT of these 45 patients and performed EWAS on the Illumina platform using the Infinium HumanEPIC BeadChip array. From the 45 patients with morbid obesity, 20 subjects were selected based on the relative abundances of the predominant phyla, Bacteroidetes and Firmicutes: high BFR (HighBFR group; BFR > 2.5; *n* = 10) vs. low BFR (LowBFR group; BFR < 1.2; *n* = 10) (**Table S2**). As expected, the HighBFR group (*n* = 10) exhibited predominance of the Bacteroidetes phylum (*p* < 0.0001), whereas Firmicutes was predominant in the LowBFR group (*n* = 10) subjects (*p* < 0.0001) (**Figure 1**). No statistical differences between other phyla such as Proteobacteria, Actinobacteria, or Fusobacteria were found between groups (**Figure 1**). The general characteristics of both study groups are summarized in **Table 1**. There were no significant differences in age, sex, and BMI between the two study groups. Glucose and HbA1c levels were significantly lower in the HighBFR group when compared with the LowBFR group (*p* < 0.05). There were no significant differences in HOMA-IR, HDL-cholesterol, and triglycerides between the two study groups (*p* > 0.05).

**Table 1:** Baseline clinical and biochemical variables of the study subjects divided by Bact/Firm ratio.

	HighBFR (*n* = 10)	LowBFR (*n* = 10)	*p* value
Age, years	42.2 ± 10.6	46.1 ± 7.2	0.353
Gender, f (%)	8/10 (80)	8/10 (80)	—
Weight, kg	144.1 ± 18.9	133.8 ± 25.5	0.796
BMI, mg/kg^2^	52.5 ± 5.4	49.4 ± 8.1	0.579
Glucose, mg/dl	93.0 ± 17.9	141.2 ± 75.3	0.015
HbA1c, %	5.7 ± 0.6	6.8 ± 2.0	0.011
HOMA-IR	6.3 ± 4.0	10.7 ± 6.6	0.089
HDL-cholesterol, mg/dl	42.5 ± 10.1	48.5 ± 9.4	0.247
Triglycerides, mg/dl	168.2 ± 111.9	147.0 ± 108.1	0.796

Data are means ± SD. p values were calculated for the difference between study groups using Mann–Whitney U test. p < 0.05 was considered significant. HOMA-IR, homeostasis model assessment of IR; HDL, high-density lipoprotein.

**Figure 2:**
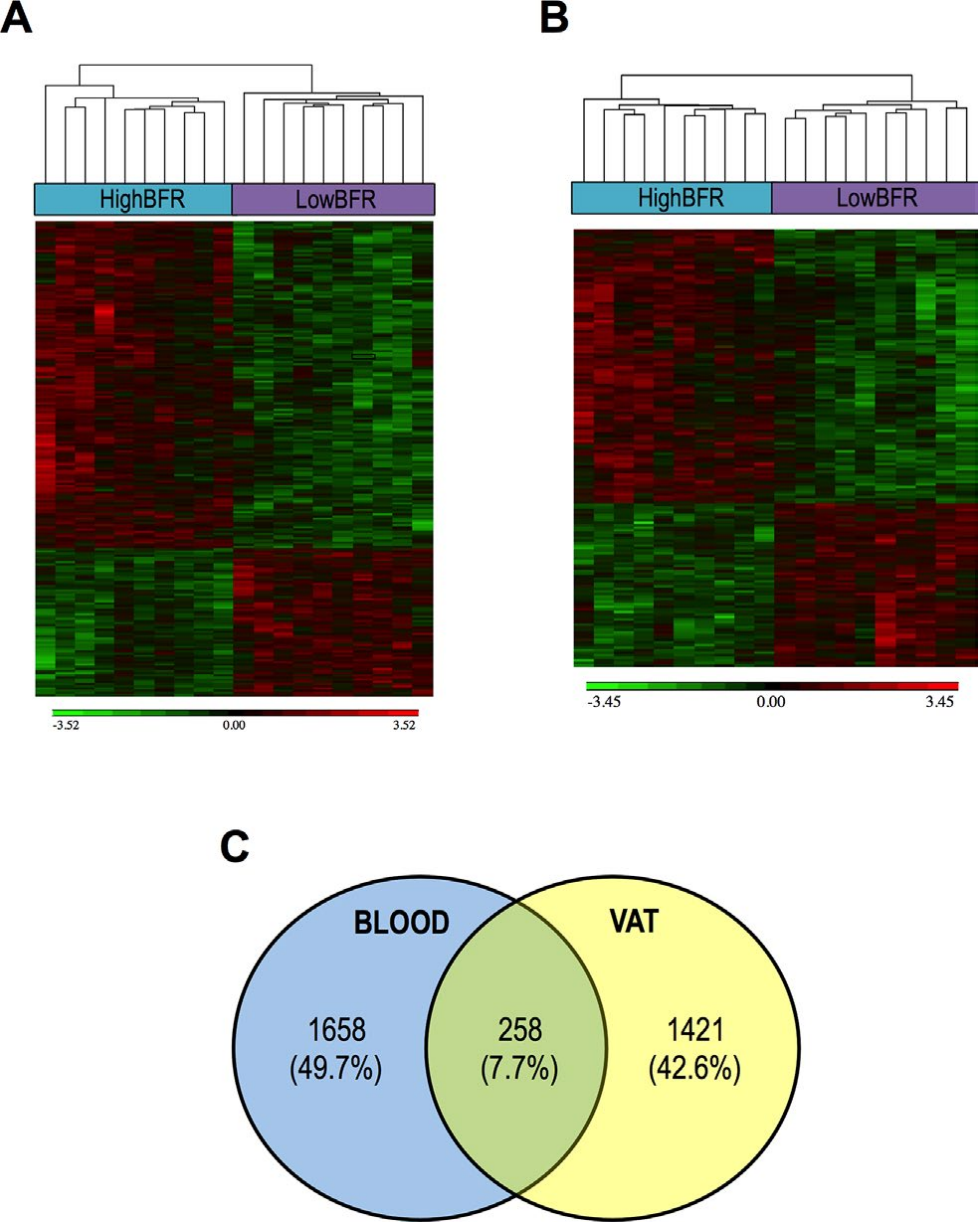
Association between the gut microbiota composition and the DNA methylome in blood **(A)** and visceral adipose tissue (VAT) **(B)**. The analysis of methylation data yielded a set of genes that were significant differentially methylated among subjects with a predominance of either Bacteroidetes (HighBFR) or Firmicutes (LowBFR) in the gut microbial population. Green indicates decreased and red indicates increased methylation in the LowBFR group compared with the promoter methylation in the HighBFR group. **(C)** Venn diagram of genes differentially methylated in both whole blood and visceral adipose tissue.

To determine the potential contribution of gut microbiota composition to the global DNA methylome, we extracted genomic DNA from whole blood and VAT and performed EWAS on the Illumina platform using the Infinium HumanEPIC BeadChip array. As shown in **Figure 2**, the two groups showed a different methylation profile in both whole blood and VAT (**Figure 2A and B**). We found 1,658 and 1,421 differentially methylated genes between study groups in whole blood and VAT, respectively (**Figure 2C**; **Tables S3 and S4**). We classified them as hypermethylated and hypomethylated, differentiating those that were significant in both whole blood and VAT from those that were only significant in whole blood or VAT (**Table S5**). Remarkably, 258 genes were differentially methylated both whole blood and VAT (**Figure 2C**; **Table S6**). Pathway enrichment analysis revealed that most of the genes differentially methylated in whole blood and VAT were involved in glycerophospholipid metabolism and cell adhesion molecules, respectively (**Table S7**). Moreover, a further enrichment analysis on an ontology basis such as OMIM diseases revealed that the top three categories enriched were related to diabetes. In order to better understand these results, a further association analysis between the probes and the phenotype characteristics of the patients was performed (**Table S8**). Many associations were found, but HOMA-IR, HbA1c, weight, and BMI were found to be the most relevant variables.

**Figure 3:**
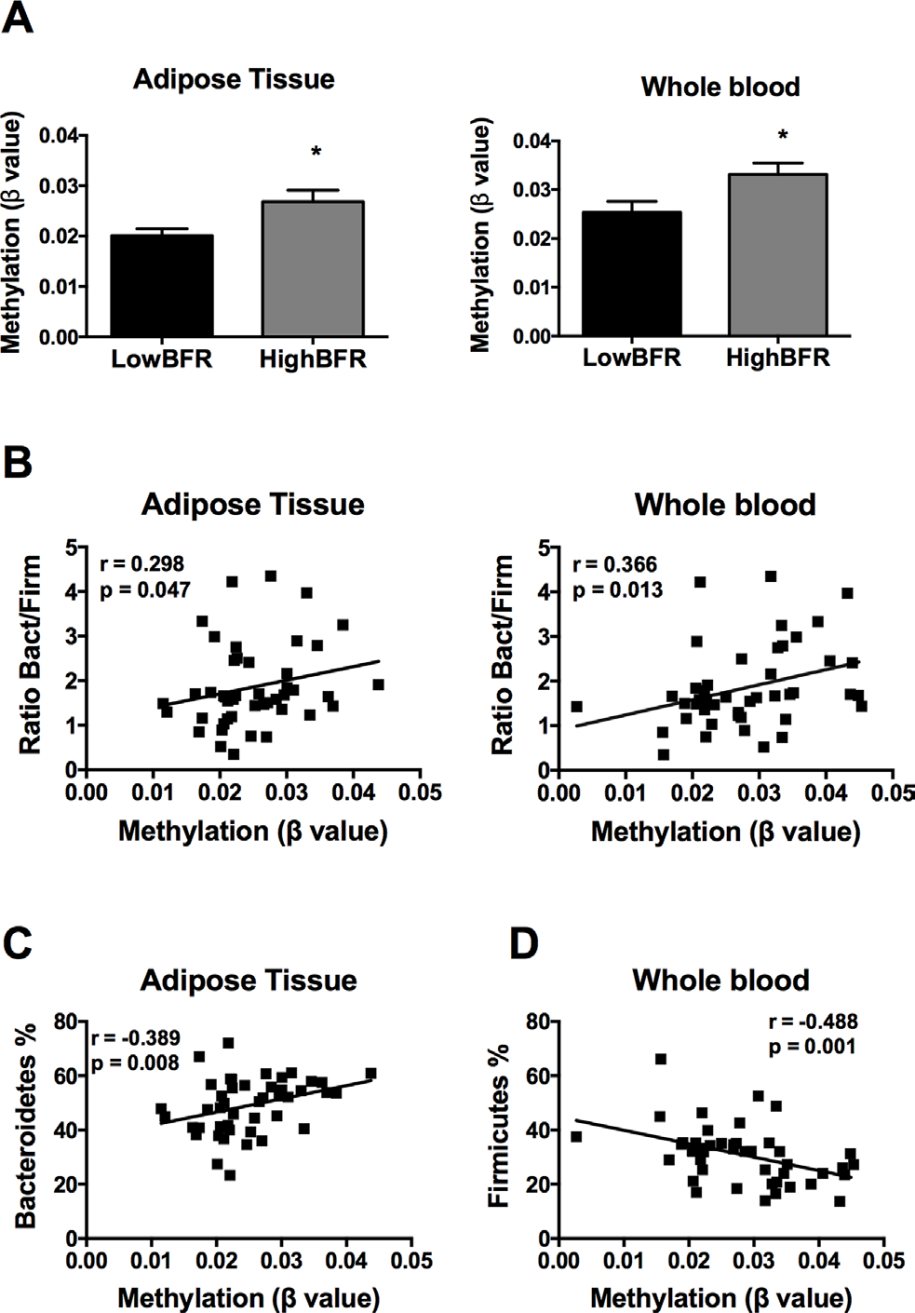
Association between the DNA methylation status of histone deacetylase 7 (HDAC7) and the gut microbiota composition in obese subjects. **(A)** Methylation of *HDAC7* (쌠β value) in the HighBFR vs. LowBFR groups in both visceral adipose tissue and whole blood. Data (*n* = 10 per group) are plotted as means ± SE. Significance was tested using Mann–Whitney *U* test and is indicated as *p < 0.05. **(B)** Spearman correlations between *HDAC7* methylation and the ratio Bact/Firm in both visceral adipose tissue and whole blood. **(C)** Spearman correlation between *HDAC7* methylation and the relative abundance (%) of Firmicutes in whole blood. **(D)** Spearman correlation between *HDAC7* methylation and the relative abundance (%) of Bacteroidetes in visceral adipose tissue.

**Figure 4:**
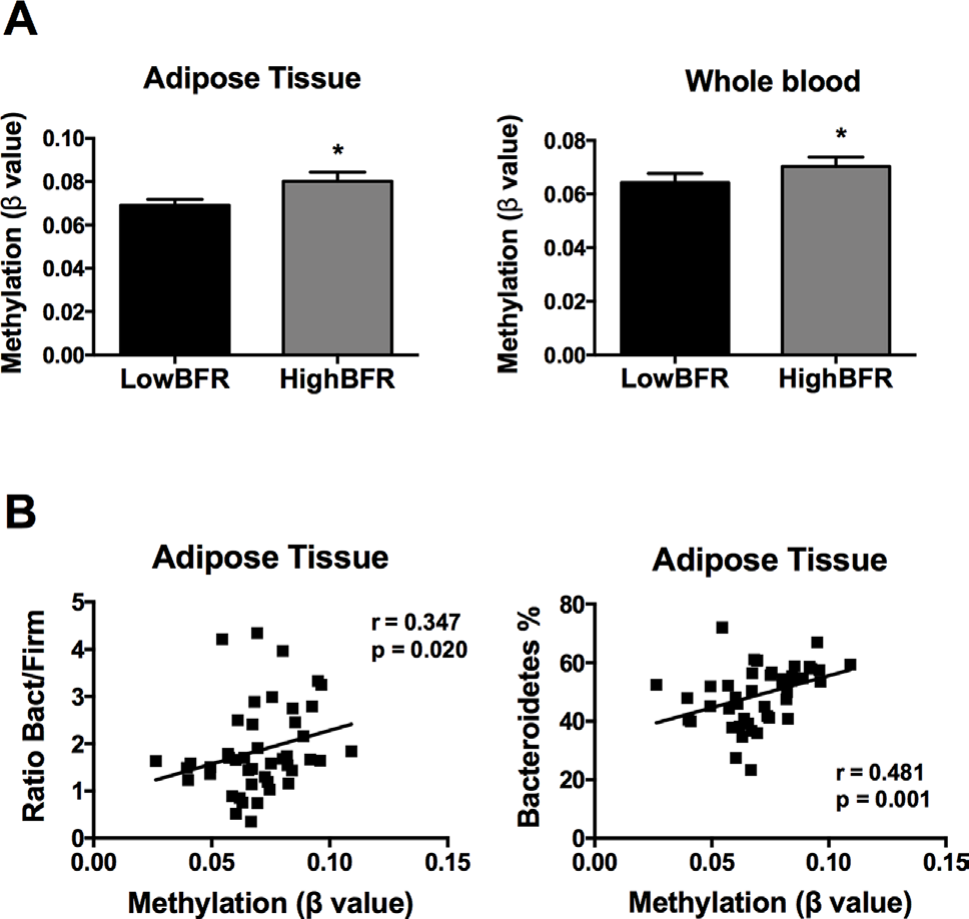
Association between the DNA methylation status of insulin like growth factor 2 mRNA binding protein 2 (IGF2BP2) and the gut microbiota composition in obese subjects. **(A)** Methylation of *IGF2BP2* (쌠β value) in the HighBFR vs. LowBFR groups in both visceral adipose tissue and whole blood. Data (*n* = 10 per group) are plotted as means ± SE. Significance was tested using Mann–Whitney *U* test and is indicated as *p < 0.05. **(B)** Spearman correlations between *IGF2BP2* methylation in visceral adipose tissue and the ratio Bact/Firm or the relative abundance (%) of Bacteroidetes.

Following an exhaustive analysis of the list of genes, we focused on genes previously related to obesity, metabolic disease, and/or T2D. Thus, we tested the impact of changes in the methylation levels on the mRNA expression levels in VAT of the following genes: *FGF1*, *FGF10*, *KDM4B*, *HDCA7*, *IGF2BP2*, *IL7*, *EDEM1*, *ATF6*, and *CDK6* (Sharma et al., 2008; Makki et al., 2013; Ohta and Itoh, 2014; Dai et al., 2015; Cheng et al., 2018; Davegardh et al., 2018; Hou et al., 2018). As shown in **Figure S1**, *HDAC7* and *IGF2BP2* mRNA levels were significantly different between study groups; no differences in the expression levels were found for the rest of the analyzed genes. Further, we assessed the association between the gut microbiota composition and the methylation levels of both *HDAC7* and *IGF2BP2*. As shown in **Figure 3A**, the β values of *HDAC7* (indicatives of the DNA methylation status of the gene) were significantly higher in the HighBFR group in both whole blood and VAT. Correlation analysis in the whole cohort of obese subjects (*n* = 45) demonstrated that the β values of *HDAC7* in both whole blood and VAT were positively associated with the BFR (**Figure 3B**). Additionally, whereas β values of *HDAC7* in blood correlated negatively with the relative abundance of Firmicutes (**Figure 3C**), β values of HDAC7 in VAT were positively associated with the relative abundance of Bacteroidetes (**Figure 3D**). Like *HDAC7*, the β values of *IGF2BP2* were significantly higher in the HighBFR group in both whole blood and VAT (**Figure 4A**). Furthermore, *IGF2BP2* β values in VAT significantly correlated with the BFR and the relative abundance of Bacteroidetes (**Figure 4B**). No significant correlation was observed between gut microbiota composition and β values of *IGF2BP2* in whole blood. It is noteworthy that some of these correlations remain still significant when patients with the most extreme BFR values were excluded (validation cohort; *n* = 25). Thus, we found that the relative abundance of Bacteroidetes positively correlated with the methylation levels of *HDAC7* (*r* = 0.500, *p* = 0.011) and *IGF2BP2* (*r* = 0.597, *p* = 0.002) in adipose tissue, and the relative abundance of Firmicutes negatively correlated with the methylation levels of *HDAC7* (*r* = −0.465, *p* = 0.019) in whole blood. These results reinforce the relationship between gut microbiota and DNA methylation within these genes.

## Discussion

Obesity is a pathological condition highly associated with lifestyle. Epigenome and gut microbiota are two factors clearly impacted by lifestyle. Recent evidence has proposed that certain metabolites produced by microbial metabolism can influence the epigenetic profile in several conditions (Hullar and Fu, 2014; Cuevas-Sierra et al., 2019). Despite the possible role of gut microbiota as epigenetic regulator, the number of works associating gut microbiome and epigenetics is scarce. Moreover, most of these studies were focused on histone acetylation, with little attention paid to DNA methylation status. Here, we have demonstrated for the first time an association between the composition of certain bacterial populations in the gastrointestinal tract with specific DNA whole-genome methylation states in both blood samples and adipose tissue biopsies in the context of extreme obesity. Overall, the subjects included in the present study were characterized by a heterogeneous gut microbiota composition. However, we found that, independently on their clinical characteristics, classification of patients clustered into two groups according to their gut microbiota profile measured by the relative abundance of the predominant phyla Bacteroidetes and Firmicutes. These clusters of obese individuals presented similar BMI and clinical parameters related to lipid metabolism but significant differences in markers of glucose metabolism. In particular, individuals with low Bact/Firm ratio displayed higher levels of fasting glucose and HbA1c. Microbiota profile is influenced by the environmental conditions (Rothschild et al., 2018). Within the gut, microbiota is influenced by the host phenotype. Gut microbiota has been extensively related to glucose levels and metabolism, although a clear conclusion about the cause or consequence has not completely been achieved (Utzschneider et al., 2016). Thus, glucose levels could drive the clusters of these patients and could influence the gut microbiota profiles and consequently the Bact/Firm ratio used in the study. The classification of obese patients according to their Bact/Firm ratio showed a clear association between the relative abundance of these phyla with the DNA methylation profile in both blood and adipose tissue, supporting the idea that the gut microbiota could act as an epigenetic regulator in obesity, as previously indicated by others for other pathological conditions (Yang et al., 2013; Hullar and Fu, 2014; Sook Lee et al., 2017; Watson and Søreide, 2017; Qin and Wade, 2018). In fact, the furthest association analysis between the DNA methylation results and the phenotypes of the patients revealed that weight and BMI, as well as HOMA-IR and HbA1c levels, were the variables more related to DNA methylation status. Interestingly, the enrichment analysis based on OMIM diseases database showed that diabetes, and particularly type 2 diabetes, was the disease most related to the DNA methylation status, which mirrored the results showed through the clustering of the patients according to their Bact/Firm ratio.

Previous studies have suggested that gut microbiota may impact the epigenetic landscape of the host. In animal models, it has been previously shown that microbial metabolites such as SCFAs can influence epigenetic programming in various tissues, including proximal colon, liver, and white adipose tissue (Krautkramer et al., 2016). Because most of butyrate-producing bacteria belong to the Firmicutes phyla (Vital et al., 2014), differences in the Bact/Firm ratio within our cohort of obese individuals could result in different circulating levels of butyrate or other SCFAs, which would explain the observed differences in the DNA methylation status in both blood cells and adipose tissue. In addition to SCFAs, other metabolites produced by the bacteria from the gastrointestinal tract have been related to epigenetic modifications (Bhat and Kapila, 2017). In particular, gut bacteria can produce high levels of folic acid and polyamines, which are molecules highly related to carbon metabolism and therefore with potential impact in the DNA methylation status (Crider et al., 2012; Soda, 2018; Ramos-Molina et al., 2019). Nevertheless, whether the changes in the methylome associated with alterations in gut microbiome are related to changes in the levels of these or other bacterial metabolites requires further investigation.

As described above, in this study, we report for the first time a possible crosstalk between the gut microbiome and the DNA methylation state in obesity. Our results are supported by multiple studies performed in cohorts of non-obese individuals. For instance, a recent pilot study performed in pregnant women demonstrated an association between the relative abundance of dominant phyla (Bacteroidetes and Firmicutes) and the DNA methylation profile in blood samples (Kumar et al., 2014). In another interesting work, Kelly et al. reported an association between gut microbiota and histone methylation signature of intestinal epithelial cells in patients with inflammatory bowel syndrome (Kelly et al., 2018). In a mouse model of diet-induced obesity, Qin et al. demonstrated that changes in the gut microbiome could result in epigenetic alterations associated with the development of colon cancer (Qin et al., 2018). There is no evidence, however, of a relationship between the composition of the gut microbiome and the methylation status in adipose tissue. Previous work from our lab demonstrated that the DNA methylation of certain genes related to adipogenesis and lipid metabolism is impaired in adipose tissue of subjects with metabolic syndrome (Castellano-Castillo et al., 2019). Nevertheless, whether these changes in the methylation pattern are related to differences in the gut microbiota composition remains unknown.

On the other hand, we have found that the promoters of both *HDAC7* and *IGF2BP2* genes were hypomethylated in whole blood and adipose tissue of the study patients with low Bact/Firm ratio. These genes were further studied based on their relationship with metabolism. However, it is worthy to mention that only two of the nine studied genes achieved a statistically significant difference between BFR groups, indicative of the complex machinery regulating gene expression and representing DNA methylation in only one of the mechanisms implicated. On the one hand, *HDAC7* gene encodes a histone deacetylase (HDAC). Histone deacetylase enzymes repress gene expression by removing an acyl group bound to chromatin. Although it is widely known that class I HDACs (mainly 1, 2, and 3) are inhibited by microbial products as SCFAs, mainly butyrate (Yuille et al., 2018), this is the first time that a class IIb HDAC is related with gut microbiota. In line with our results, a previous work demonstrated that the *HDAC7* gene was hypomethylated and overexpressed in islets from donors with T2D (Dayeh et al., 2014), which could have pathological implications given that *Hdac7* overexpression in rat islets and β-cell lines resulted in impaired insulin secretion (Daneshpajooh et al., 2017). Our results show that hypomethylation in the *HDAC7* promoter in both whole blood and adipose tissue is also associated with disturbances in glucose metabolism, as both study groups displayed marked differences in glucose and HbA1c levels. This suggests that the changes in the methylation profile in the *HDAC7* gene are related not only to the composition of the gut microbiota but also to the metabolic profile of the subjects, at least in blood and adipose tissue. However, further investigation is required to examine in detail the implication of the microbial population.

On the other hand, hypomethylation of *IGF2BP2* also resulted in higher mRNA levels in adipose tissue. In adipose tissue, *IGF2BP2* is able to downregulate the expression of *IGF2*, a growth factor that plays a pivotal role in controlling adipogenesis (Louveau and Gondret, 2004). Therefore, impaired *IGF2BP2* expression levels may contribute to the development of metabolic disorders such as obesity and T2D through alterations in the function of the adipose tissue. In this regard, inactivation of the *IGF2BP2* in mice induces resistance to diet-induced obesity and fatty liver due in part to increased energy expenditure, suggesting that *IGF2BP2* has an important role in the regulation of energy homeostasis (Dai et al., 2015). Thus, gut microbiota profile could be participating in the homeostasis of the host through the methylation of particular genes as *IGF2BP2*. Interestingly, these associations between gut microbiota and *HDAC7 and IGF2BP2* gene expression and methylation levels seem to be driven by the phylum Bacteroidetes. The major end products of Bacteroidetes are succinate, acetate, and, in some cases, propionate (Chakraborti, 2015). Methylation rates depend on the availability of one- and two-carbon substrates (Su et al., 2016). Acetate is a two-carbon substrate, while succinate is able to follow the tricarboxylic acid cycle. Thus, although classically Firmicutes has been the main phylum related to epigenetic modifications, Bacteroidetes could be more related to methylation and Firmicutes to acetylation modifications. However, phylum is a phylogenetic level that groups different microbial members with different SCFAs and other metabolites that should be carefully studied.

In conclusion, we demonstrate that the methylation status could be largely affected by the gut microbiota composition in obese subjects and that the expression levels of genes implicated in glucose and energy homeostasis (e.g., *HDAC7* and *IGF2BP2*) could be epigenetically regulated by gut bacterial populations in adipose tissue. In order to understand how gut microbiota can influence DNA methylation in adipose tissue and other target organs, further studies are needed.

## Data Availability Statement

All datasets generated for this study are included in the manuscript and the supplementary files.

## Ethics Statement

All participants provided written informed consent, and the study protocol and procedures were approved according to the ethical standards of the Declaration of Helsinki by the Research Ethics Committees from the participating institution (Virgen de la Victoria University Hospital, Malaga, Spain)

## Author Contributions

BR-M, IM-I, and FT designed research. BR-M, LS-A, AC-M, RL-D, and PC-S conducted research. EG-F provided essential materials. BR-M and IM-I analyzed the data. BR-M, IM-I, and FT wrote the paper. BR-M, IM-I, and FT had primary responsibility for the final content. All authors read and approved the final manuscript.

## Funding

This study was supported by the “Centros de Investigación Biomédica en Red” (CIBER) of the Institute of Health Carlos III (ISCIII) (CB06/03/0018) and research grants from the ISCIII (grant numbers PI15/01114 and PI18/01160) and co-financed by the European Regional Development Fund (ERDF). BR-M was a recipient of a Sara Borrell postdoctoral fellowship from the ISCIII (CD16/0003) and co-funded by the ERDF. IM-I was supported by the Miguel Servet Type I program (CP16/00163) from the ISCIII and co-funded by the ERDF.

## Conflict of Interest Statement

The authors declare that the research was conducted in the absence of any commercial or financial relationships that could be construed as a potential conflict of interest.

## Supplementary Material

Table S1 Baseline clinical characteristics of study subjects.

Table S2 Relative abundance of bacterial phyla in fecal microbiota.

Table S3 List of genes differentially methylated in whole blood.

Table S4 List of genes differentially methylated in visceral adipose tissue.

Table S5 Genes differentially hypomethylated and hypermethylated in both whole blood and visceral adipose tissue.

Table S6 List of genes differentially methylated both in whole blood and visceral adipose tissue.

Table S7 Pathway analysis.

Table S8 Association analysis between methylation levels and the phenotype characteristics of the study patients.
