# Supplementary material for: Gut Microbiota Composition Is Associated With the Global DNA Methylation Pattern in Obesity
Source: Front Genet. 2019 Jul 3;10:613. doi: 10.3389/fgene.2019.00613 (PMC6616130; doi:10.3389/fgene.2019.00613)
Supplement: Supplementary file 9 [file Image_1.pdf]

Supplementary Material

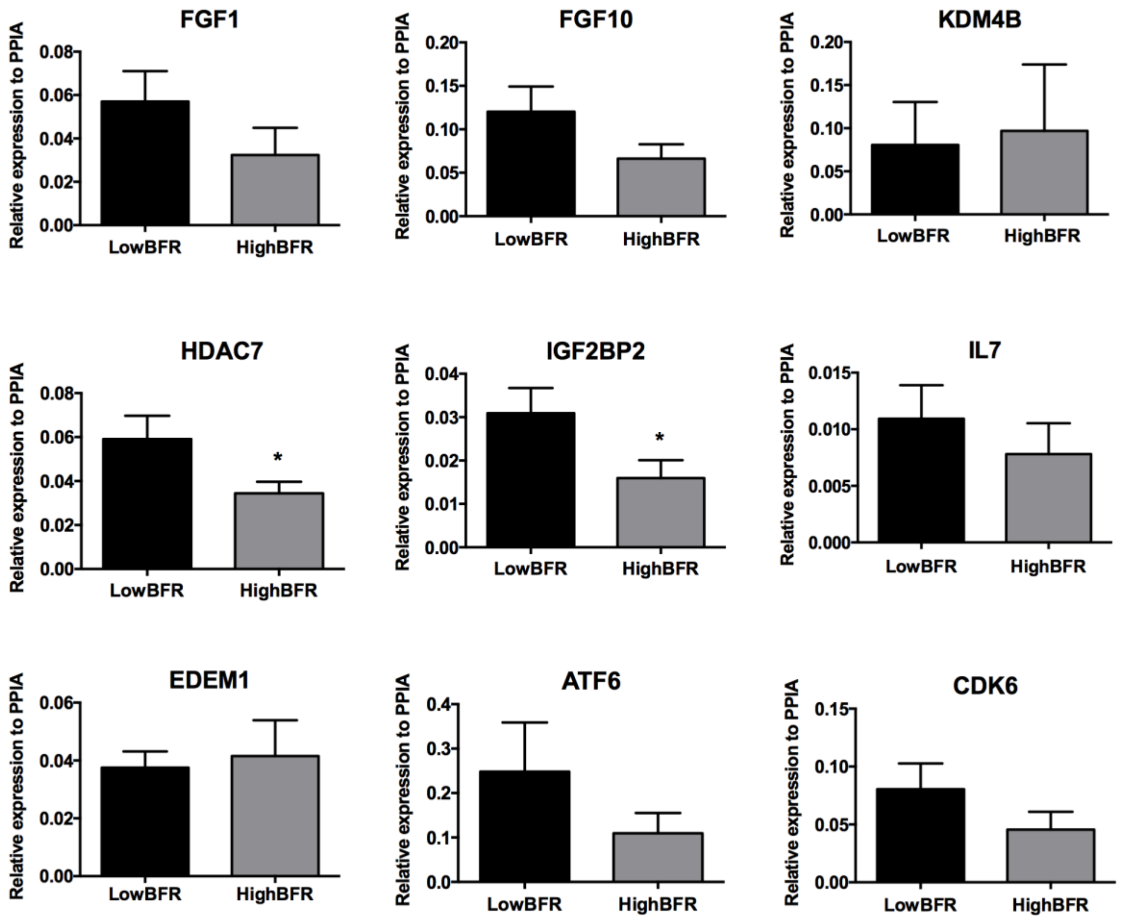

Supplementary Figure 1. Expression levels in adipose tissue of selected genes with differentially methylated regions.
